# Analysis of the Properties of Modified Asphalt Binder by FTIR Method

**DOI:** 10.3390/ma15165743

**Published:** 2022-08-19

**Authors:** Chi-Su Lim, Dae-Sung Jang, Sang-Min Yu, Jae-Jun Lee

**Affiliations:** Department of Civil Engineering, Jeonbuk National University, 567 Baekje-daero, Deokjin-gu, Jeonju-si 54896, Jeollabuk-do, Korea

**Keywords:** asphalt binder, Fourier transform infrared spectroscopy (FTIR), ethylene-vinyl acetate (EVA), styrene–butadiene–styrene (SBS), warm additives

## Abstract

The usage of modified asphalt binder in road pavements has been increasing in the past few decades. Therefore, quality control and understanding of modified asphalt binders have become an important issue. This study was conducted as a part of a larger study on the efficient management of these modified asphalt binders by evaluating the characteristics of asphalt binders mixed with styrene–butadiene–styrene (SBS), ethylene-vinyl acetate (EVA), and wax-based warm-mix additives using Fourier transform infrared (FTIR) analysis. For original asphalt binders modified with SBS, response wavenumbers were 700 and 966 ${\mathrm{cm}}^{-1}$, which means a particular wavenumber of polybutadiene and polystyrene, while in the case of binders modified with EVA, peak response wavenumbers were at 1242 and 1739 ${\mathrm{cm}}^{-1},$ which represents a particular wavenumbers of a single stretching bond between carbon and hydrate and a double stretching bond between carbon and oxygen. Asphalt binders modified with wax-type additives showed peak response at 730 and 1540 ${\mathrm{cm}}^{-1},$ which represents a double stretching bond of carbon and a single stretching bond between nitrogen and oxygen. It was also found that peak values increased as addition rates also increased. The results showed that the additives used in this study have particular wavenumbers that show peak responses even when mixed into asphalt binders. Using these characteristics of the additives, FTIR analysis confirms that it is possible to determine whether or not a binder has been modified.

## 1. Introduction

Asphalt pavements consist of aggregate, asphalt binder, and air voids; they are a viscoelasticity material affected by loading time and temperature. Generally, asphalt binder is a complex material composed of carbon, hydrogen, nitrogen, and sulfur [1]. The aging of asphalt binder is one of a number of major factors that cause defects, including cracks, scaling, and plastic deformation in the pavement [2]. The aging of asphalt, which is closely related to service life, occurs in response to changing chemical properties, temperatures, and ultraviolet rays over time [3]. Commonly, asphalt binder consists of asphaltene, which provides structural stability, and maltene, which affects strength and ductility. As aging progresses, asphaltene and maltene content declines, and these changes in chemical composition make the asphalt mixture stiff and brittle [4]. The aging process of asphalt can be divided into two categories: short-term aging, which occurs during the storage and construction period, and long-term aging, which occurs during service life. When a pavement’s surface layer comes into direct contact with traffic load during its service life, aging causes the asphalt binder to harden, which induces defects such as labeling and cracking [5]. Since demand for asphalt pavement roads continues to increase in spite of dwindling supplies of industrial resources, a number of studies have been conducted around the world on methods of extending the pavement life cycle and reducing resource and maintenance costs. These studies have developed a wide range of materials that improve the properties of asphalt, including polymer additives, rejuvenators, and fiber. Of the various methods that improve the properties of asphalt mixtures, additives are currently the most frequently used method [6]. Styrene–butadiene–styrene (SBS) is a polymer that is widely used to improve the plastic deformation resistance and low-temperature crack resistance of asphalt pavement and has been studied in numerous countries for some time [7]. A wide range of studies have been and continue to be conducted that compare the properties of asphalt binders modified by SBS with those of original binders. In one case study, a creep test was conducted on mixtures with SBS-modified asphalt binder and one without SBS, and the results confirmed that the SBS-modified mixture has excellent low-temperature sensitivity and compact ability compared to HMA [8]. Ethylene-vinyl acetate (EVA) is a plastomer obtained by mixing ethylene and vinyl acetate that is used to modify asphalt binders. The properties of EVA vary depending on the content of vinyl acetate. However, due to problems such as phase separation between polymer and binder, one case study examines storage stability and mixing conditions in relation to the mixing of EVA and asphalt binder [9]. In addition to these polymeric asphalt additives, many other studies are being conducted on warm-mix additives, which can mitigate environmental pollution by reducing the production and construction temperature of the asphalt mixture as a solution to low carbon emission and high oil prices [10]. Among other research cases, through dynamic shear rheometer (DSR) and bending beam rheometer (BBR) tests to evaluate the properties of warm-mix asphalt binders using oxidized polyethylene wax and siloxane types of additives, differences of various kinds were discovered between the properties of the two additives, such as softening properties [11]. Recently, many studies have been conducted based on chemical and rheological properties as well as physical properties due to the aging of asphalt binders. Linear amplitude sweep (LAS) test was conducted to evaluate the rheological properties of asphalt binders according to the laboratory aging level, FTIR test was conducted to evaluate the carbonyl index of aged binder, and the obtained results had a high correlation [12]. In addition, in order to improve the properties of the existing warm-mix additives, gilsonite was added together with sasobit (a warm-mix additive) for further analysis using FTIR, DSR, surface free energy (SFE), etc. In the FTIR analysis, the addition of gilsonite did not produce any peak response, and most of the peak values were the same, meaning there was no chemical reaction; therefore, the use of a composite of gilsonite and sasobit could improve the thermomechanical properties of the binder [13]. The use of FTIR in the chemical analysis of binders has been diversified, and there are cases where FTIR was applied to analyze reclaimed asphalt binder (RAP). In one study on the blending characteristics of virgin asphalt binders and RAP asphalt binders, a blending chart, which relates to RAP binder content and carboxyl indexes of blended binder was established, and diffusion of virgin binder into RAP can be evaluated through a scanning electron microscope (SEM) and energy dispersive X-ray spectroscopy (EDS) analysis [14]. In another study that was conducted FTIR analysis for microanalysis of asphalt using as a modifier, the natural rock asphalt modifier was added to penetration 70 asphalt binder, and the characteristics for short-term and long-term aging were evaluated. When 6% of natural rock asphalt was added, the carbonate index and the sulfoxide index were the lowest, and the aging resistance was the best [15]. Many studies are currently being conducted on these various types of asphalt binders, and in the long term, extending the service life of asphalt can be achieved through effective quality control of asphalt binders. However, most asphalt plants currently have been conducting a penetration test or absolute viscosity test as a quality control method for binders, which varies in the result value due to the proficiency of the tester. Therefore, it is necessary to develop a more scientific approach to the asphalt binder quality control method. This study thus investigates one method of managing the quality of asphalt binders by evaluating the properties of asphalt binders mixed with various types of additives using FTIR analysis.

## 2. Materials

### 2.1. Polymer Modified Asphalt Binder Using Styrene–Butadiene–Styrene (SBS)

Polymer is a material that can improve the properties of asphalt binder and extend the service life of asphalt pavement, and styrene–butadiene–styrene (SBS) is the most widely used type of polymer in asphalt pavements. SBS increases viscosity and improves both elasticity recovery and strain resistance due to its elastic properties [16]. In addition, SBS-modified binders have excellent resistance to fatigue cracks and plastic deformation compared to unmodified binders [17]. Because of these properties, polymer-modified asphalt binders are used in locations with high traffic volume conditions, and their proven superiority in terms of plastic deformation resistance, stripping resistance, and thermal cracking resistance makes them an important resource in the pavement industry [18]. Asphalt binders used were graded using a Superpave performance grading system which is based on the climate (high and low temperature) in which the pavement will serve. In this study, asphalt binders with performance-grade PG 82-22 and PG 76-22 were modified for the experiments using SBS.

### 2.2. Polymer Modified Asphalt Binder Using Ethylene-Vinyl Acetate (EVA)

Ethylene-vinyl acetate (EVA) is a polymer-based thermoplastic material similar to low-density polyethylene (LDPE) and plasticized polyvinyl chloride (PVC) [19]. EVA is one of the most representative materials used to produce polymer-modified asphalt (PMA), the same as SBS or styrene–butadiene–rubber (SBR). When EVA was mixed into epoxy asphalt, which is normally used for bridge deck pavement, it increased viscosity and improves thermal stability and mechanical properties through phase changes inside the material [20]. In this study, FTIR analysis confirmed inherent absorbance peaks in EVA and changing peaks when mixed with PG 64-22 (original binder).

### 2.3. Warm-Mix Asphalt Additives

Until recently, most pavements have been constructed using hot mix asphalt, but the use of warm-mix asphalt is increasingly common nowadays because of budget restrictions and environmental conservation. Warm-mix asphalt reduces energy emissions by lowering the temperature during asphalt mixture production [21]. In addition, depending on the location of a plant, the transportation distance and construction period of asphalt mixtures can be reduced while also shortening opening times for traffic. Recently, various types of additives for warm-mix asphalt have been developed [22]. Based on previous research, the binders were mixed with two types of warm-mix wax additives. A wax-type additive capable of lowering a heating temperature of a general asphalt binder by lowering the viscosity of an asphalt binder was referred to as L-Wax, and the other one which improved rutting resistance of the asphalt binder due to a polymer component was referred to as H-Wax. To fabricate warm mix asphalt mixture, both L-Wax and H-Wax were used. Normally, the mixing ratio of L-Wax and H-Wax was 3:1, and Table 1 shows the mixing ratios of modified asphalt binder by warm-mix additives used for analyzing absorption peak changes. 

## 3. Methodology

### 3.1. Sample Preparation

**Figure 1:**
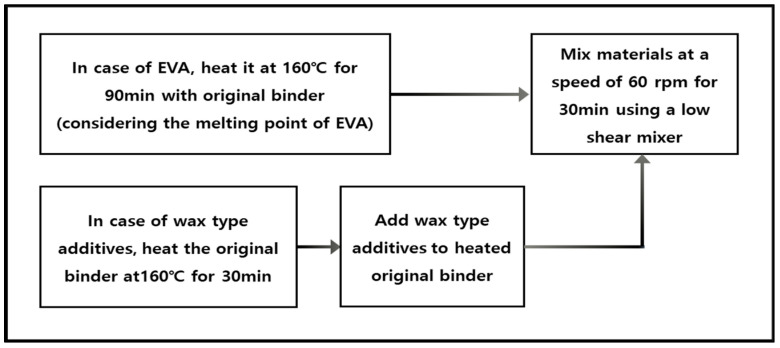
Production process for modified asphalt binders.

The study focused on analyzing different types of modified binders and their characteristics using FTIR. Various types of additives were used. For FTIR test, four different types of modified asphalt binder were used. Two types of modified asphalt binder were PG 82-22 (PMA) and 76-22 (PMA) modified by SBS, a commercial polymer. The other modified binders used to analyze the rate of addition of warm-mix additives and to evaluate EVA-modified binders were produced by the process shown in Figure 1.

### 3.2. FTIR (Fourier Transform Infrared) Analysis

**Figure 2:**
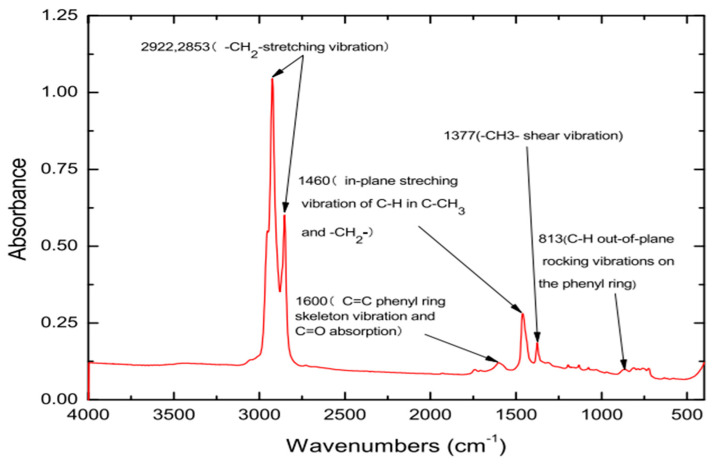
FTIR spectrum of original asphalt.

**Figure 3:**
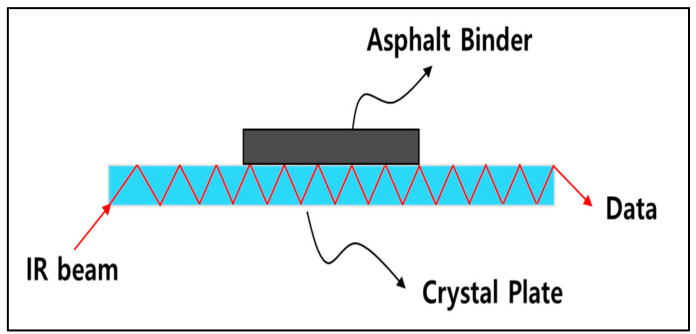
Principle of FTIR-ATR.

FTIR analysis is a measurement method based on the principle that the molecules that make up a given material molecules structure absorb energy or radiation of a defined wavenumber. FTIR is widely used in many industries related to chemical bonding, such as medicine and food, because it is better suited for the analysis of molecular structure more than any other analytical technique. Typical analysis techniques include the diffuse reflectance inspection (DRIFTS) technique, which analyzes powdered type substances, and the roughing angle inspection specification (RARS) technique, which requires thin film and detailed sample preparation [23]. In the case of asphalt, more than 90% of the material consists of a combination of carbon and hydrogen, and since its form depends on the degree of oxidation, this can be evaluated through FTIR analysis [24]. When analyzing original binders, FTIR analysis shows peaks at wavenumbers of 2922 ${\mathrm{cm}}^{-1}$, 2853 ${\mathrm{cm}}^{-1}$, 1600 ${\mathrm{cm}}^{-1}$, 1460 ${\mathrm{cm}}^{-1}$, 1377 ${\mathrm{cm}}^{-1}$, and 813 ${\mathrm{cm}}^{-1}$, as shown in Figure 2 [25]. In this study, the FTIR device PerkinElmer L160000A from the USA was used in all experiments. Samples were directly attached to the crystal surface of the device, and attenuated total reflectance (ATR) mode was applied as shown in Figure 3. ATR applied is unaffected by the shape and color of the samples measured. In FTIR analysis, one result was obtained by calculating the average value after scanning each sample 10 times, and a total of 3–5 repetitive analyses were conducted on the same sample by varying the sampling positions.

## 4. Results and Discussion

### 4.1. Comparison of FTIR Absorption Peak by Additives

**Figure 4:**
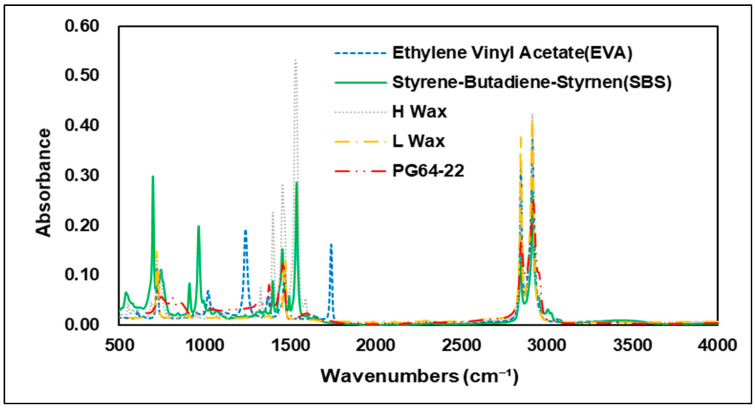
FTIR spectrum of asphalt binder additives.

**Figure 5:**
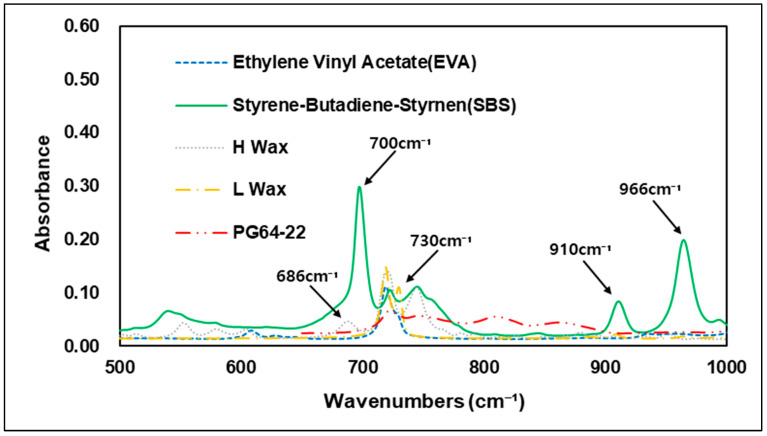
FTIR Spectrum of 500–1000 cm^−1^ area.

**Figure 6:**
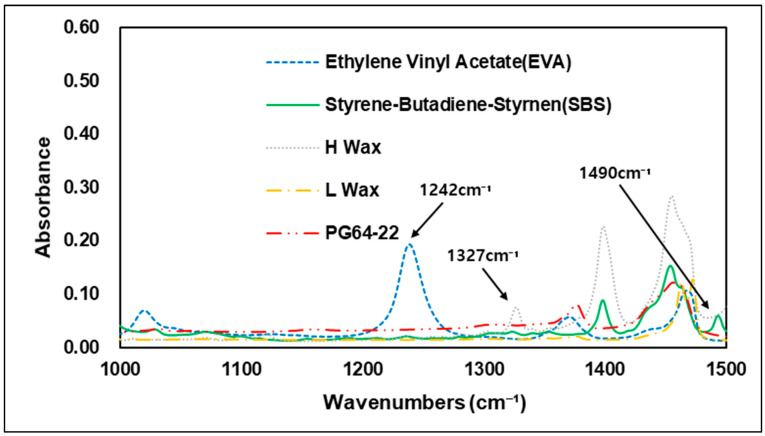
FTIR Spectrum of 1000–1500 cm^−1^ area.

**Figure 7:**
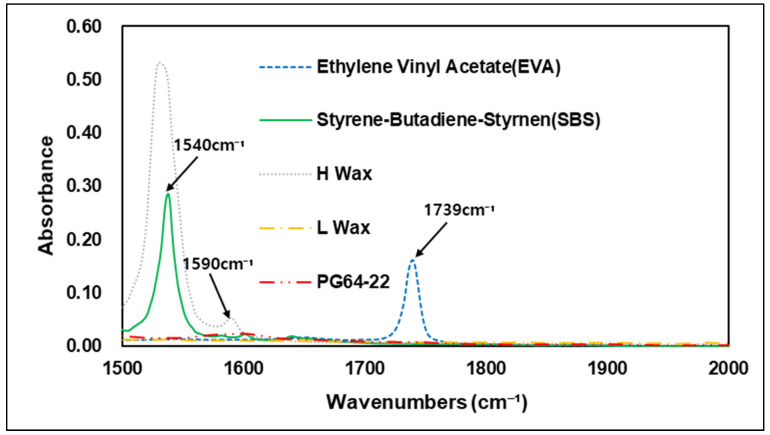
FTIR Spectrum of 1500–2000 cm^−^¹ area.

**Figure 8:**
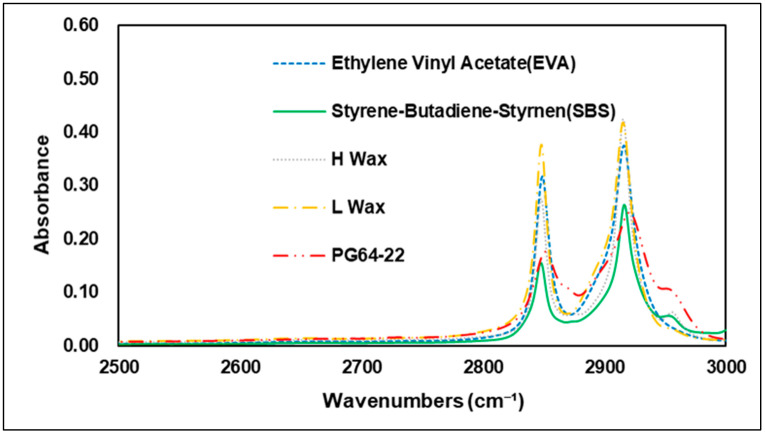
Spectrum of 2500–3000 cm^−^¹ area.

Various types of additives have been developed to improve the performance of asphalt. These additives have the advantage of improving resistance to rutting, low-temperature cracking of asphalt pavement, which can extend the service life of a pavement [26]. Styrene–butadiene–styrene (SBS) is used primarily in modified asphalt binders, and it has advantage in high plastic deformation resistance at high temperatures through improved temperature sensitivity [23]. In FTIR analysis, carbon bonds C=C are usually utilized to evaluate the characteristics of SBS. The 1450 ${\mathrm{cm}}^{-1}$ and 2920 ${\mathrm{cm}}^{-1}$ wavenumbers show peaks of carbon–hydrogen bonding (C-H), but the best characteristics of SBS are shown at the 966 ${\mathrm{cm}}^{-1}$ and 700 ${\mathrm{cm}}^{-1}$ wavenumbers, which represent polybutadiene and polystyrene [27]. In addition to SBS and styrene–butadiene–rubber (SBR), a number of studies worldwide have also been conducted on Waste Tire Rubber (WTR), and EVA, which are recycled waste materials thus reduce environmental pollution when used with asphalt binders. Related studies have confirmed that when EVA is added to a modified asphalt binder, it is peak-responsive in wavenumber of 1243 ${\mathrm{cm}}^{-1}$ and 1740 ${\mathrm{cm}}^{-1}$ by the combination of stretching vibration of C-H and vibration of C=O bonds [28]. Hot mix asphalt typically consumes a large amount of energy and releases greenhouse gases, such as CO_2_ when aggregates and binders are heated at temperatures above 140 °C [29]. Warm-mix asphalt was first introduced in Europe in the late 1990s. Since then, with the development of various materials, it has been possible to achieve adequate workability and compaction with lower energy consumption due to warm-mix asphalt’s lower mixing temperature compared to conventional HMA. In addition to reducing energy consumption, warm-mix asphalt also has the advantage of reducing the construction period and extending the carry distance of asphalt mixtures [30]. In the United States, standardization studies have been carried out since 2002, when the technology was first introduced, and the National Center for Asphalt Technology (NCAT) developed quality and performance certification processes for the warm-mix asphalt mixture in 2008 [31]. A number of studies of warm-mix asphalt mixtures also have been conducted in the Republic of Korea, and use of WMA is gradually increasing. Many types of additives are currently used in the civil engineering industry, depending on the purpose required. The present study was conducted to determine the different chemical properties of these additives. Using FTIR analysis, the specific wavenumbers of each additive were evaluated and compared with the original binder. Figure 4 shows the wavenumbers of the additives used in the study. Experiments showed that each of the additives had an absorption reaction and a number of absorptions in different regions that varied depending on the additives in question. Similar to previous studies, SBS showed absorption reactions in the C=C bond and the C-H bond, with peaks in the 700, 910, 966, 1490, and 1540 ${\mathrm{cm}}^{-1}$ wavenumbers, while EVA showed peaks in the 1242 and 1739 ${\mathrm{cm}}^{-1}$ wavenumbers, which are related to C-H and C=O bonds. The warm-mix additives of H-wax type showed peak response in the wavenumbers of 686, 730, 1327, 1540 ${\mathrm{cm}}^{-1}$ and 1590 ${\mathrm{cm}}^{-1}$, while in L wax, most peak areas were found to overlap with the H-wax and original asphalt binder. In overlapping regions, L-wax was confirmed to have lower absorbance peaks than H-wax and the original asphalt binder. This means that modifying the asphalt binder using the warm-mix additives H-wax and L-wax can be determined based on the particular wavenumbers of the H-wax. Figure 5, Figure 6, Figure 7 and Figure 8 are graphs grouped by wavenumbers at regular intervals to confirm the peak of a particular wavenumbers more easily. The graph shows that the wavenumbers at peak response for each of the additives and the original asphalt binder are different, which means that properties of chemical bonds can be determined when additives with particular wavenumbers are mixed with an asphalt binder to assess what kind of additives were used. Table 2 summarizes wavenumbers with peak responses for each additive.

### 4.2. FTIR Analysis of Modified Asphalt Binder

**Figure 9:**
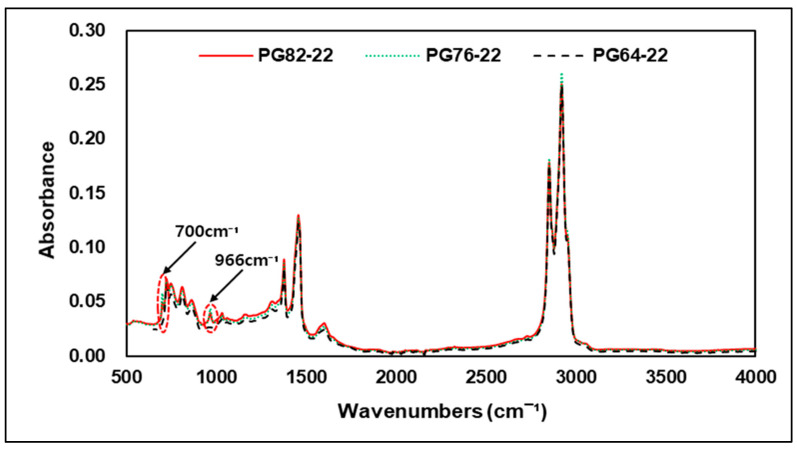
FTIR spectrum of PG 82-22, PG 76-22, and PG 64-22.

**Figure 10:**
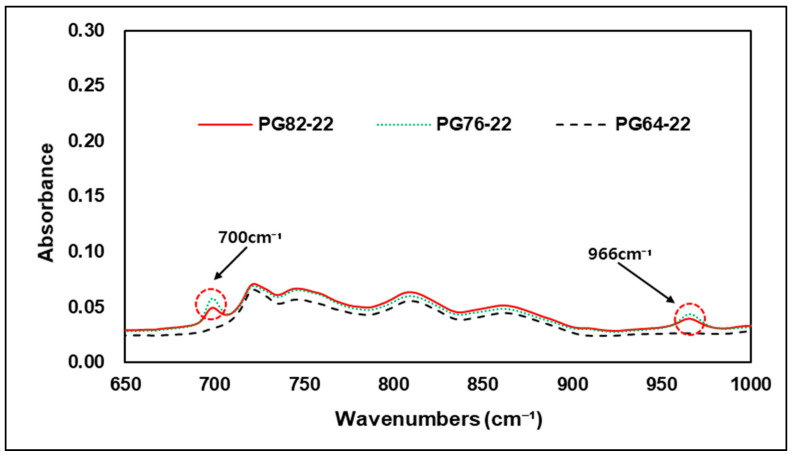
FTIR Spectrum of 650–1000 ${\mathrm{cm}}^{-1}$ area.

This study was conducted to verify the detectability of the additives discussed earlier in an asphalt binder using FTIR analysis. Figure 9 shows the results of FTIR analysis of different asphalt binders and original binders PG 82-22 and PG 72-22 modified by SBS. As shown in the graph, the SBS-modified binder did not show bands at 1490 ${\mathrm{cm}}^{-1}$ and 1540 ${\mathrm{cm}}^{-1}$ wavenumbers representing deformation and stretching vibrations of the C-H bond, but the wavenumbers of 700 and 966 ${\mathrm{cm}}^{-1}$ representing polybutadiene and polystyrene showed a peak response compared to the original asphalt binder. This means that when SBS and asphalt binders are mixed together, the chemical reactions of modified binders cannot be detected at all wavenumbers indicating peak reactions of SBS and that the improvement of the high-temperature performance of asphalt binders with SBS is related to particular wavenumbers 700, 966 ${\mathrm{cm}}^{-1}$ of modifiers. Therefore, through the wavenumbers of the C=C bond, the binder with an improved high-temperature performance by SBS can determine whether it’s modified or not. Figure 10 shows an enlarged graph of the wavenumbers of the carbon bond referred to earlier to facilitate the identification of peak responses in specific regions of the SBS-modified binder.

**Figure 11:**
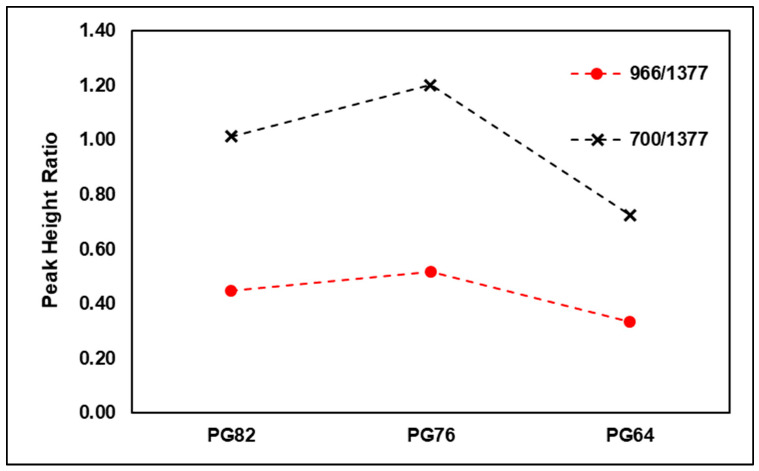
Peak height ratio by PG grade.

Furthermore, the additive content of PMA can be roughly inferred by deriving the ratio of additives’ particular peak heights to the particular peak height of the original asphalt binder [32]. In this study, the ratio of the SBS peak point was derived by setting the particular wavenumber of the asphalt binders to 1377 ${\mathrm{cm}}^{-1}$ in accordance with previous cases. As shown in Figure 11, both types of SBS-modified binders of PG 82-22 and PG 76-22 were found to have higher peak rates compared to the original binder measured in the particular wavenumber. However, binders of PG 82-22, which are expected to have higher SBS content, were found at a lower peak rate compared to binders of PG 76-22. This phenomenon was assumed to be due to the oxidation reaction of a binder exposed to air during the manufacturing process, as well as the non-homogeneous mixing of the binder with the additives.

**Figure 12:**
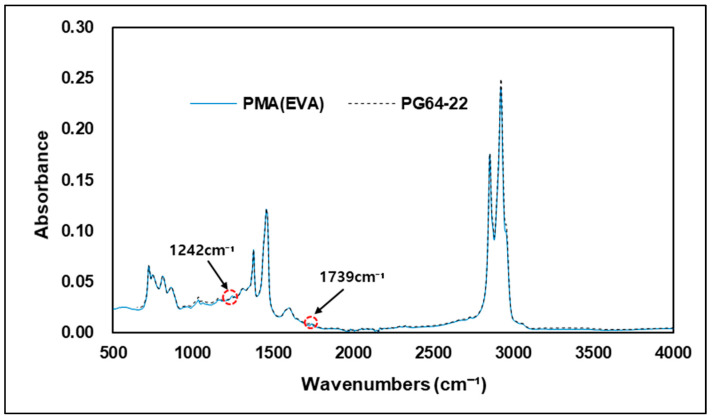
FTIR spectrum of PMA (EVA) and PG 64-22.

**Figure 13:**
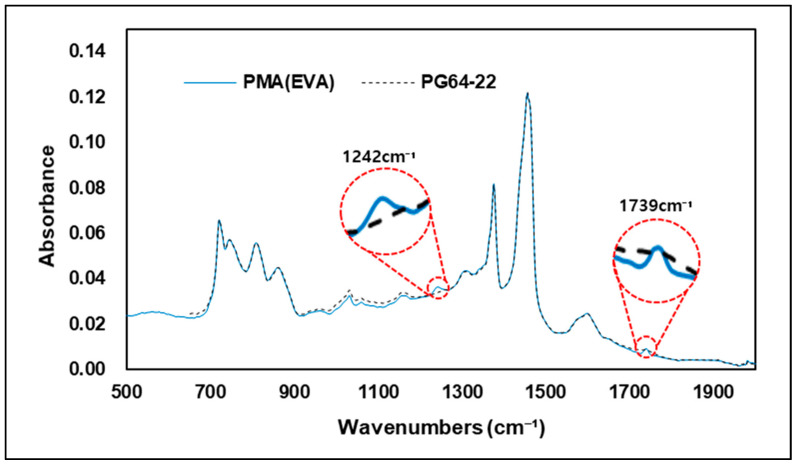
FTIR spectrum of 500–2000 cm^−^¹ area.

Figure 12 shows the FTIR spectrum between the original asphalt binder and the EVA-modified binder. Results from FTIR analysis show that the EVA 4% modified binders and the original asphalt binder have similar peak responses across the entire wavenumbers. However, as shown in Figure 13, at 1242 ${\mathrm{cm}}^{-1}$ and 1739 ${\mathrm{cm}}^{-1},$ which represents the C-H bond and C=O bond, the particular wavenumbers of EVA, only modified binders showed a slight peak response. These reactions were in both wavenumbers previously observed as particular regions of EVA, meaning that absorption reactions in these wavenumbers can determine whether a binder was modified or not using EVA. However, outside these two wavenumbers, it would be difficult to ascertain whether a binder is EVA-modified or not using FTIR alone. This is due to the similarity of the peak reactions to those of the original binder. The results of this experiment are for the modified binder with 4% EVA added, and it is necessary to review the change in the peak response when the modified binder is produced by increasing or decreasing the amount of EVA added. In addition, although not confirmed in this study, it is also necessary to investigate peak responses that may occur due to new chemical bonds in the wavenumbers of EVA-modified binders that showed peak reactions similar to original binders when the asphalt is modified by adding EVA at a higher level from 4%.

**Figure 14:**
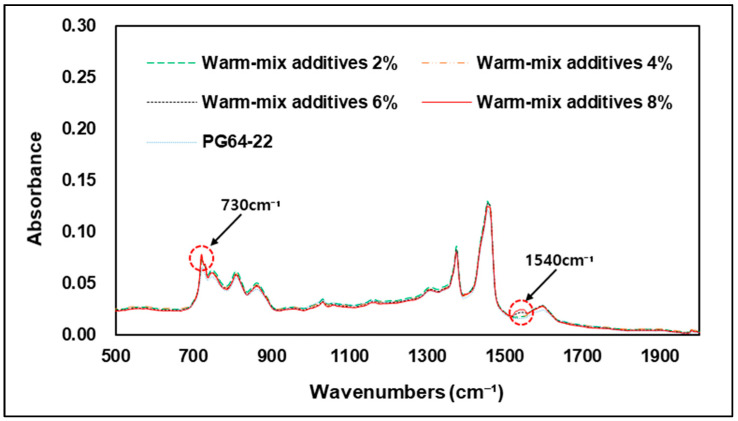
FTIR spectrum by warm-mix additives contents.

**Figure 15:**
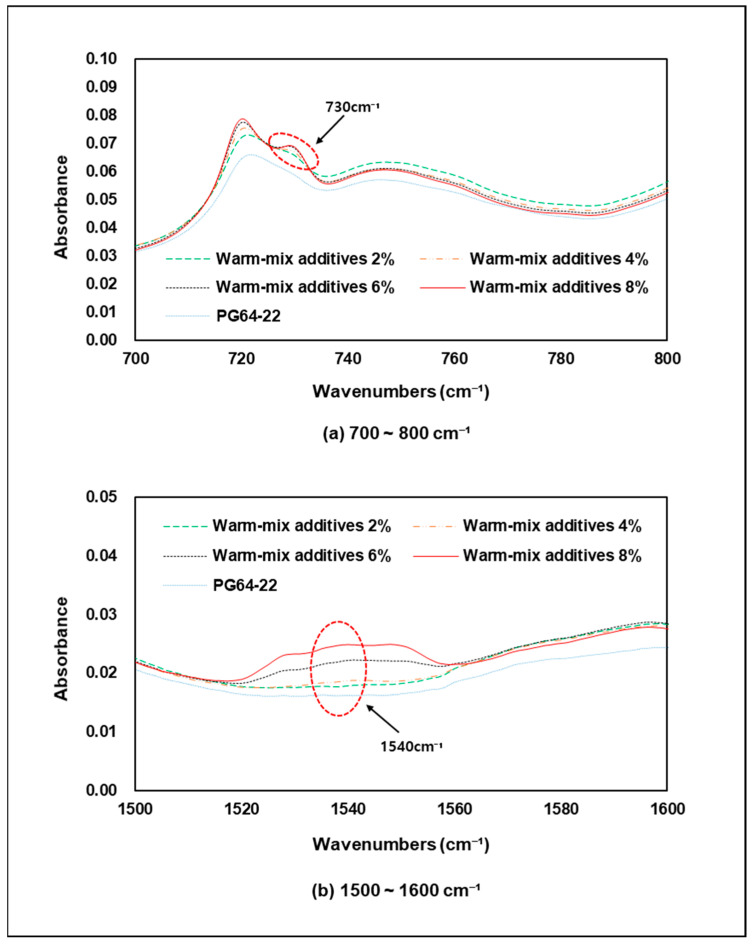
Peak wavenumber of warm-mix additives modified asphalt binder.

Figure 14 shows the results of FTIR analysis by addition rates of warm-mix additives. As previous modifiers analysis, the warm-mix additive showed peak response at wavenumbers such as 686, 730, 1327, 1540, and 1590 ${\mathrm{cm}}^{-1}$, but when mixed with the original binder, it was found that the peak response was wavenumbers only at 730 and 1540 ${\mathrm{cm}}^{-1}$. Particularly, in the case of L wax, the level of response detected through FTIR analysis is very low, and the wavenumber is similar to the original binder, so it is difficult to check whether L wax is added in the modified asphalt binder using FTIR. Therefore, it was decided that the modified asphalt binder with warm-mix additives of the wax type used in this study should be detected at 730 ${\mathrm{cm}}^{-1}$ wavenumber, which C=C bond means a double stitching bond between carbon and 1540 ${\mathrm{cm}}^{-1},$ which means a single stretching bond between nitrogen and oxygen, as shown in Figure 4. Figure 15 shows an enlarged graph of the partial wavenumbers of the modified binder according to the addition rate of the warm-mix additives, and it can be seen that the peak responses at 730 ${\mathrm{cm}}^{-1}$ and 1540 ${\mathrm{cm}}^{-1}$, increase as the addition rate increases. This means that the bond of C=C and N-O in the asphalt binder increases as the addition rate of warm-mix additive increases, and that it is possible to calculate the addition rate of additives through FTIR analysis in the future. However, wavenumber at 1540 ${\mathrm{cm}}^{-1}$, the lower the warm-mix additive rate, the lower the peak response was, and at addition rates of 2% and 4% showed a very small reaction compared to the original binder, and the clear peak response was when the addition rate was 6% and more. Therefore, when small amount of additives are added, it will be difficult to detect whether or not the binder is modified using warm-mix additive at 1540 ${\mathrm{cm}}^{-1}$ wavenumber.

**Figure 16:**
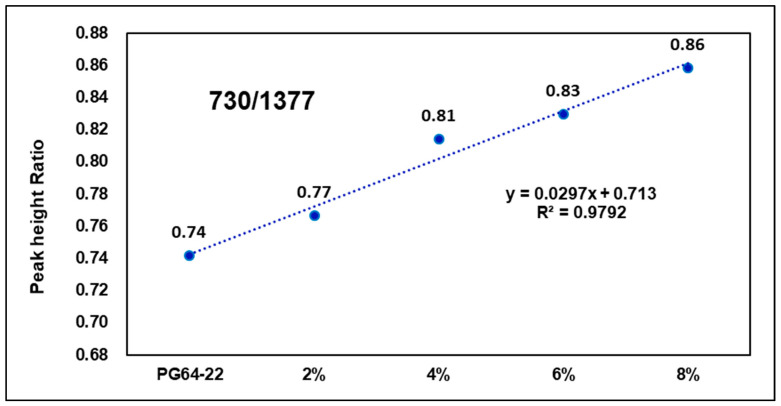
Warm-mix additives modified binder peak ratio (730/1377).

**Figure 17:**
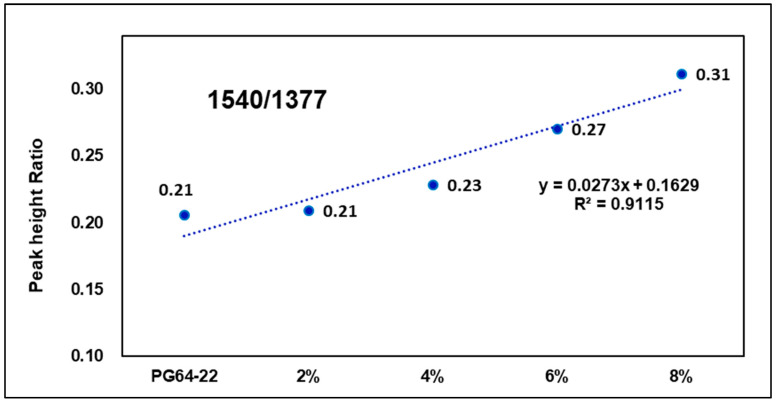
Warm-mix additives modified binder peak ratio (1540/1377).

Figure 16 and Figure 17 show peak height ratios of particular wavenumbers for binders with different warm-mix additives addition rates and the original binder. As seen in the graphs, as warm-mix additives content increased, the peak height ratio also increased. The wavenumber 730 ${\mathrm{cm}}^{-1}$ tended to have a steeper slope than 1540 ${\mathrm{cm}}^{-1}$, as well as a higher coefficient of determination. This is because, as the warm-mix additives rate increased, the peak response at 730 ${\mathrm{cm}}^{-1}$ increased at a constant rate, but as below the addition rate of 4%, the peak response at 1540 ${\mathrm{cm}}^{-1}$ did not have a clearly increasing trend. As a result of the experiment, FTIR analysis of warm-mix asphalt mixture was able to determine which type of warm-mix additives have been used; also, further studies should be conducted on developing new methods for more accurately calculating additive content, as well as other types of warm-mix additives.

## 5. Conclusions

This study was conducted to evaluate the properties of asphalt binders modified with several types of additives using FTIR analysis. Its overall conclusions are as follows:

1. SBS-modified asphalt binders with improved performance showed peaks at 700 ${\mathrm{cm}}^{-1}$ and 966 ${\mathrm{cm}}^{-1}$ wavenumbers, compared with non-peaks from the original asphalt binder. This means that FTIR analysis can detect whether an asphalt binder is SBS-modified or not.
2. FTIR analysis of EVA-modified binders revealed that SBS and EVA showed almost identical peaks but at wavenumbers of 1242 ${\mathrm{cm}}^{-1}$ and 1739 ${\mathrm{cm}}^{-1}$, where only EVA-modified binder responded. However, these peak responses were not significant for binders modified with EVA amounts of less than 4%, from which it is infrared that they are difficult to detect by FTIR analysis.
3. According to FTIR analysis of binders modified with warm-mix additives, the peaks appear to be in the 730 ${\mathrm{cm}}^{-1}$ and 1540 ${\mathrm{cm}}^{-1}$ regions, but the differences in peak values were not significant between the original binder and binders modified with addition rates of 2~4%, but the addition of more than 6% showed a significant difference.
4. This study was conducted to evaluate differences between modified and original binders using FTIR analysis. It was concluded that FTIR analysis constitutes a viable identification method for asphalt binders modified by additives for properties improvement. Further research is needed on other types of additives, as well as to assess the field applicability of FTIR analysis by investigating chemical bond changes under aging conditions.

## Figures and Tables

**Table 1:** Mixing ratios of modified asphalt binder by warm-mix additives.

Total Warm-Mix Additives Content (%)	0	2	4	6	8
L Wax Weight (g)	0	7.5	15	22.5	30
H Wax Weight (g)	0	2.5	5	7.5	10
PG 64-22 Binder Weight (g)	500	490	480	470	460

**Table 2:** Particular wavenumbers of each additive.

Type of Additives	Wavenumbers (cm^−1^)				
Styrene–butadiene–styrene (SBS)	700	910	966	1490	1540
Ethylene-vinyl acetate (EVA)	1242			1739	
Warm-mix asphalt additives (WAX)	686	730	1327	1540	1590

## Data Availability

Not applicable.
